# Adverse events with ayurvedic medicines- possible adulteration and some inherent toxicities

**DOI:** 10.12688/wellcomeopenres.15096.3

**Published:** 2019-11-01

**Authors:** Buddhi Paudyal, Astha Thapa, Keshav Raj Sigdel, Sudeep Adhikari, Buddha Basnyat

**Affiliations:** 1 Department of Internal Medicine, Patan Academy of Health Sciences, Lalitpur, Nepal; 2 Oxford University Clinical Research Unit, Patan Hospital, Lalitpur, Nepal

**Keywords:** Ayurvedic medicine, heavy metals, alkaloids, adulteration

## Abstract

Ayurvedic medicine, a traditional system of medicine practiced in the Indian subcontinent is considered to be devoid of adverse events. We report three cases which highlight the possibility of adverse events related with the use of ayurvedic products. A 35 year old woman with hepatitis took ayurvedic powder medicine and swarnabhasma (gold salt) and had her liver injury worsened, possibly due to alkaloids, and developed nephrotic syndrome, possibly due to gold salt. A 57 year old hypertensive man was taking ayurvedic medicine containing reserpine which had long been withdrawn from the allopathic system of medicine due to wide range of side effects. A 47 year old woman with rheumatoid arthritis was taking an unknown tablet containing steroid as an adulterant for 2 years and developed side effects typical of steroid excess. We would like to highlight the fact that ayurvedic medicines do have propensity to cause adverse events due to adulteration or inherent constituents like alkaloids, and hence may not always be completely safe.

## Background

Ayurveda is one of the most renowned traditional systems of medicine, and has been widely practiced in the Indian subcontinent, including Nepal, since the 2 ^nd^ century BC ^1^. People have faith with ayurveda as it is based on the use of natural products, and is considered to be devoid of adverse events ^2^.

Allopathic medicines on the other hand are known to have adverse events, and are generally prescribed based on risk versus benefit for a particular disease and patient ^3^. Alternative forms of medicine like ayurveda are usually thought by patients and ayruvedic doctors alike to be harmless, and are also advertised similarly ^2^. But it has been proven that certain constituents of ayurvedic products, like heavy metals and alkaloids, can have adverse events, and the possibility of these adverse events needs to be highlighted so that both the practitioners and consumers will become cautious in their use, as with allopathic medicines ^4, 5^.

Sometimes, patients receive unknown powders adulterated with drugs such as steroids in the name of ayurvedic medicine, prescribed by traditional healers ^5^. People’s faith in ayurvedic medicines has been exploited by many healers who prescribe such unnamed powders to patients, especially with chronic diseases like arthritis and asthma, leading to adverse events.

## Case 1

**Figure 1.**
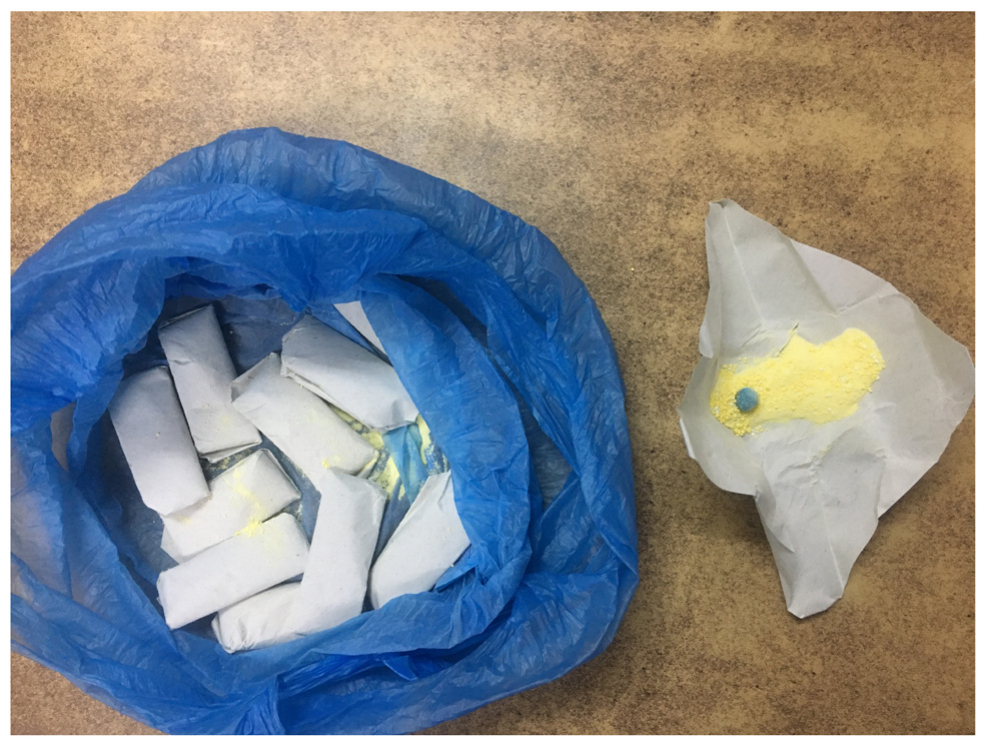
‘Puriya’ containing powder medicines taken by case 1.

A 35 year old Newar woman from suburban Kathmandu who was a housewife, developed jaundice, vomiting and low grade fever. Family members took her to a local healer who claimed to be an ayurvedic practitioner. He prescribed a combination preparation called “puriyas” in paper packets containing several powder medicines ( Figure 1) and gold salt (swarnabhasma). Despite the treatment, she became sicker with deepening of jaundice and significant weight loss (almost half of her previous body weight) in about one week. She was then rushed to the emergency department (ED) of Patan Hospital, Lalitpur, Nepal (April, 2018). On presentation to ED, her laboratory parameters, with normal range in parantheses, were as following;

Complete blood count (CBC): white cell count 10.9 (4–10) × 10 ^9^/L; neutrophils 70%; lymphocytes 22%; monocytes 8%; red blood cells 4.8 (4.2–5.4) × 10 ^12^/L; haemoglobin 12.1 (12–15) g/dL; platelets 136 (150–400) × 10 ^9^/L.

Biochemistry: random blood sugar 123 (79–160) mg/dL, urea 59 (17–45) mg/dL; creatinine 1.3 (0.8–1.3) mg/dL; sodium 138 (135–145) mmol/L and potassium 4.3 (3.5–5) mmol/L

Hepatic panel: bilirubin total 65.73 (0.1–1.2) mg/dL and direct 43.9 (0–0.4) mg/dL; alanine transaminase (ALT) 566 (5–30) units/L; aspartate transaminase (AST) 494 (5–30) units/L; alkaline phosphatase (ALP) 155 (50–100) IU/L; albumin 3.0 (3.5–5) g/dL, International normalized ratio (INR) 2.0 (0.9–1.2)

Urine examination: albumin 3+, sugar- nil, white cell count 1–2/ high power field, red blood cells- nil, 24 hour urine protein- 3.5 gm/ day

Viral hepatitis panel: Hepatitis A virus (HAV) IgM, Hepatitis E virus (HEV) IgM, HBsAg, Hepatitis C virus (HCV) IgM- all negative

She was admitted and diagnosed as fulminant hepatic failure with possible infective hepatitis and nephrotic range proteinuria. The powder was stopped, and she was managed with supportive treatment (intravenous fluids, intravenous ceftriaxone 1 gm and oral doxycycline 100 mg twice daily for 7 days, daily blood glucose and alternate day hematology, electrolytes, renal and hepatic biochemistry monitoring). She was discharged from hospital in two weeks after she started improving. She recovered, with bilirubin and transaminases falling gradually to normal after three weeks (bilirubin-total 1.0 mg/dL and direct 0.6 mg/dL, ALT 30 units/L and AST 23 units/L). Her proteinuria also decreased gradually (24 hour urine protein- 0.8 gm/day), and urine dipstick for protein was negative at one month.

## Case 2

A 57 year old gentleman from Kathmandu, a teacher by occupation had come for a blood pressure check-up at the medical outpatient department (OPD) of Patan Hospital, Lalitpur, Nepal in June, 2018. He told the doctor that he had been taking an ayurvedic medicine called “Tensarin” for high blood pressure for the past 3 years. The composition leaflet revealed that this drug contained several herbal preparations, one of which was “ *Rauwolfia serpentina*” from which the active substance “Reserpine” is derived. His blood pressure during this visit was 140/80 mm Hg. Reserpine is not a recommended agent for treating hypertension due to its adverse events such as decreased cardiac output, bradycardia, sedation, depression, diarrhea, and increased gastric acid. Fortunately, our patient had no adverse events attributable to reserpine. We explained to him the risks of the drug he was receiving, then switched him to amlodipine 5 mg once daily. He has been in regular follow up now and his blood pressure continues to be well controlled.

## Case 3

**Figure 2.**
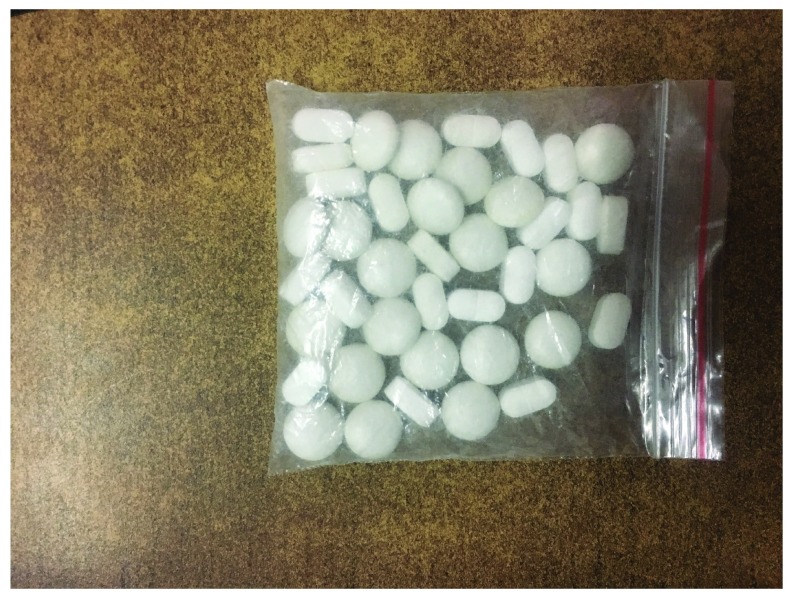
Unlabeled tablet taken by case 3.

A 47 year old Aryan woman from suburban Lalitpur who was a housewife, came to the OPD of Patan Hospital in April, 2018 with a complaint of excessive weight gain. She claimed that she was gaining excessive amount of weight despite maintaining her normal diet and physical activity. She also had sore muscles and bruises on her body. On further inquiry, she said that she had been experiencing pain over multiple joints for past 2 years. She was taking some unlabeled tablet ( Figure 2) prescribed to her by a local practitioner whom she believed to be an ayurvedic doctor. The tablet controlled her pain, and it also made her feel “strong” as she could perform her chores that she was unable to, prior to that medication. On further questioning as to why she chose this form of medication, she said that she believed the ayurvedic medicine were potentially harmless. The drug was sent for chemical analysis, and it revealed that the tablet consisted of prednisolone, an exogenous steroid. Soon after we stopped the unlabeled medicine, she started to have more pain and swelling in the small joints of both hands and symptoms suggestive of steroid withdrawal were noted. She was subsequently diagnosed as rheumatoid arthritis based on clinical features and laboratory parameters: C- reactive protein- 25 mg/L (normal < 5 mg/L), Rheumatoid factor- 30 IU/mL (normal < 25 IU/mL). She was then managed with disease modifying anti-rheumatic drugs (DMARDs); oral methotrexate 7.5 mg per week for 2 weeks followed by 15 mg per week thereafter and oral hydroxychloroquine 400 mg once daily. Low dose steroid (oral prednisolone 20 mg daily) was continued with gradual taper and stopped over 3 months. When last seen 3 months ago (October, 2018), her arthritis was well-controlled with DMARDs without steroid preparations or unlabeled medicines.

## Discussion

In the first case, the patient with jaundice due to infective hepatitis was taken directly to an alternative medicine practitioner. Jaundice is a problem which most of the Nepalese people in general, regardless of the status of their education, consider as a disease requiring alternative medicine such as ayurvedic medicines. Even if they consult allopathic clinicians first, many take ayurvedic medicines after a period of time as it usually takes many days to weeks for jaundice to resolve; and their faith in ayurvedic medicines gets strengthened. But unfortunately, many plant products contain alkaloids such as pyrrolizidine which are toxic to the liver, and may precipitate or worsen liver injury ^4^. In our patient, it was not clear whether the medicine she received initially contained harmful alkaloids as the analysis was not done. It was also not clear whether the worsening of her liver disease was caused by the disease process itself or the use of the powder medicine, but we can at least say that these products were not helpful in dealing with her liver problem as is generally believed. We see many patients coming to our clinic with worsened jaundice after the intake of ayurvedic products.

Our first patient also received gold in the form of swornabhasma that has been linked with kidney injury and nephrotic syndrome ^6^. Ayurvedic products contain certain amounts of different heavy metals like gold, lead, mercury, copper, iron, arsenic, zinc, and cadmium that are believed to have therapeutic benefits. Studies have shown that most of the ayurvedic products contain these metals in amounts exceeding WHO permissible limits, and can potentially cause harmful consequences to human health ^7^. The possibility of the potentially toxic amount of these heavy metals should also be considered and precautions taken. So it is imperative that the amount of such constituents in each of the ayurvedic products be mentioned on the label so that patients have some idea of the substance and the quantity being taken.

Our second patient had been taking reserpine in the form of ayurvedic product, as an antihypertensive agent. Though there are studies demonstrating the safety and efficacy of reserpine use as an antihypertensive agent ^8, 9^, the present allopathic guidelines do not support the use of such adrenergic inhibitor as a first line treatment for hypertension, nor is it included among any of the indications for treating hypertension 10. Reserpine causes depletion of norepinephrine, thereby producing adverse events such as decreased cardiac output, bradycardia, sedation, depression, diarrhea, and increased gastric acid. Its wide range of side effects led to the limitation of its use in allopathic system of medicine several decades ago 11, but it is still used in the ayurvedic system. Moreover, the product Tensarin did not have the exact amount of reserpine and other constituents mentioned in its label. So it was risky to continue the drug without knowing the exact amount the patient was receiving.

Adulteration of ayurvedic products has been another alarming issue ^12, 13^. We see many patients with arthritis and asthma like our third patient, coming to our clinic with classic Cushingoid character and many of the other adverse events of chronic steroid usage: hypertension, weight gain, hyperglycemia, osteoporosis, bone fracture, muscle weakness, ocular effects, gastrointestinal effects, and electrolyte imbalance ^14^, following years of taking adulterated ayurvedic products. Adrenal crisis when stopping these drugs is a potential problem.

Most people in South Asia believe that ayurvedic products are safer and more effective for chronic diseases ^2^. Patients with chronic disease are more vulnerable to mishaps related to ayurvedic products because they want to get rid of their chronic disabling condition, and tend to try alternative medicine products in the hope of safety and cure. Many of these chronic diseases (diabetes, hypertension, arthritis, cancer) may have no cure in either allopathic or alternative medicine practices, and patients should be counselled regarding this fact so that expectations are realistic.

## Conclusion

Ayurvedic medicines may be beneficial to health, but are not devoid of adverse events which may be due to adulteration or some inherent constituents like alkaloids. Each chemical compound in any ayurvedic preparation should be listed in the manufacturer’s label along with the amount which may lead to proper dosing, and may reduce adverse events. Proper counselling by health professionals, especially regarding adverse events, will play an important role in minimizing harm.

## Consent

Written informed consent for publication of clinical details and clinical images were obtained from the patients.

## Data availability

All data underlying the results are available as part of the article and no additional source data are required.
