# Supplementary material for: Adverse events with ayurvedic medicines- possible adulteration and some inherent toxicities
Source: Wellcome Open Res. 2019 Nov 1;4:23. Originally published 2019 Feb 6. [Version 3] doi: 10.12688/wellcomeopenres.15096.3 (PMC6790910; doi:10.12688/wellcomeopenres.15096.3)
Supplement: Peer review history

# Peer review: Reviewer response for version 2

**DOI:** 10.21956/wellcomeopenres.16814.r36496

**Published:** 2019-10-10

**Authors:** Phaik Yeong Cheah

**Affiliations:** 1 Mahidol-Oxford University Tropical Medicine Research Unit, Faculty of Tropical Medicine, Mahidol University, Bangkok, Thailand

Thank you for asking me to review this interesting paper. My review is brief as most of the issues have been addressed by the other reviewers. In addition, although I have training in pharmacy and pharmaceutics, I do not have expertise in ayurvedic medicine.

 My main comment is regarding terminology.  

 The term “adverse effects” is used by many but it is not an official FDA/ICH GCP term. If the authors would ike to use this term, please provide definition and reference.

 “Adverse events”, “adverse drug reaction (ADR)”  - please see definition provided by ICH GCP and FDA guidelines. The term side effect is often used instead of “adverse drug reactions”. ADR implies that there is a causality whereas adverse events does not.

 Eg. see Conclusion.

 Ayurvedic medicines may be beneficial to health, but are not devoid of **adverse effects. Adverse events** may be due to adulteration or some inherent constituents like alkaloids.

 Adverse effects and adverse events are not the same thing.

  Please check for typos and grammatical errors – eg. in the abstract

 Typo  “35 years old woman” should be “35 year old woman” etc

 We like to highlight – we would like to highlight

 Finally, for the Discussion section, do the authors have any recommendations or research agenda?

 Thank you

I confirm that I have read this submission and believe that I have an appropriate level of expertise to confirm that it is of an acceptable scientific standard.

# Response

**Published:** 2019-10-30

**Authors:** Sudeep Adhikari

**Affiliations:** Patan Academy of Health Sciences, Nepal

Thank you for the review.

 Corrections of typos and grammatical mistakes have been done in the revised version, especially in the abstract section.

 The term "adverse events" has been used instead of "adverse effects".

# Peer review: Reviewer response for version 2

**DOI:** 10.21956/wellcomeopenres.16814.r36106

**Published:** 2019-08-29

**Authors:** Kshirod Kumar Ratha

**Affiliations:** 1 National Research Institute of Ayurvedic Drug Development, Kolkata, West Bengal, India

1. Coming to the contents and case reports mentioned in this article, review on this article would be lengthier than the article itself.
2. The first sentence / paragraph of the abstract / introduction need refinement. Ayurveda never considered to be devoid of adverse effects. The classics have vividly explained possibilities of adverse effects and their management systematically.
3. Further, many statements need to be re-written. Ayurveda never used alkaloids exclusively in their formulations. Alkaloids are not sole but, a part of the formulation. The concept of Ayurveda drug formulation is entirely different than as understood by the authors in the manuscript. 
4. The first case have taken *puriyas* (possibly a packaging system that dispensed medicines in a paper wrapping) from a self-claimed Ayurvedic healer, which contained *Swarna bhasma*.
  1. The medicine was only given in a paper wrapping. In such case, how it was ascertained that the *puriya* contain *Swarna bhasma*. The medicine was not subjected to any analysis in order to claim they had *Swarna bhasma* in toxic levels.
  2. Previous disease history or reports are not considered to check the condition of patient prior to medications. When she visited the so called Ayurveda physician; already she was presenting with some liver pathology. In such case, how to confirm that the manifestation is because of *puriyas*. Further, how many days the *puriyas* were consumed, what were the doses, how they were administered is unclear.  
  3. One doesn't or should not become a Ayurvedic healer just by claiming.
  4. Gold is claimed to be toxic by the authors by citing a reference. It is fine for that specific instance. Processed Gold ( *Swarna bhasma*) is entirely different than the Gold that is being highlighted by the authors. Please refer: Paul and Sharma (2011) ^1^ and Jamadagni *et al*. (2015) ^2^ for further needful information.
5. The 2 ^nd^ case: No clear mentioning regarding whether the "TENSARIN" was taken by patient under prescription or by OTC. Withdrawing the drug was a good decision, but patient was put on allopathic medicine again though his Blood Pressure was near normal to his age. (Lin et al. (2016) ^3^. Claiming to have achieved normo-tension when patient was already normal with previous medications need attention. Reserpine as an extract is the culprit in unregulated dose, but blame has been put on *Sarpagandha* and formulations containing *Sarpagandha*. It is unclear (from the discussion) whether the patient is taking reserpine or Sarpagandha? What is being used in the formulation? Again, the drug regulating mechanisms existing in the country need a serious retrospective analysis here.
6. The 3rd case again talks about some unidentified traditional healer whom the patient believed as an Ayurveda physician.
7. The conclusion given by the article is unrealistic as they opine to mention all the CHEMICAL COMPOUNDS present in a formulation. Even the authors mention of **probability** of adverse events by the prior medications, but in my opinion until the causation is not fully established, reporting and blaming a medicine / medical system is unethical. 
8. These cases are treated and reported in Nepal? If, so how the practices are regulated there. It is a matter of series concern. In India, the system is regulated at different levels. The administration at Nepal should take a serious note on such practices and develop a stringent mechanism.
9. The discussion highlighted upon alkaloids like - pyrrolizidine and possibility of hepatic impairment. Which medicine from the three cases being reported in the manuscript contain pyrrolizidine? Hypothetical predictions harm systems.
10. Authors themselves are expressing that the patients/sufferers are wasting time by running here and there for satisfactory relief. In such cases, it is better generating awareness and implement stringent drug regulating and implementing practicing policies. Blaming a system will not yield anything.

 

 Ayurveda concepts of drug administration are quite different than the conventional approaches. Besides other basic requirements; understanding digestive ability, metabolic capability, tolerability of the patient to a specific dose of the drug, psycho-somatic constitution etc. of the patient is essential before starting treatment. The observations made in the article appears to be illogical and based on inadequate data. They need a further thorough review.

 A drug can be panacea or poison. Drugs fulfilling the criterion of a standard drug will always become panacea provided, if it is used properly. On the other hand, a poorly prepared or manufactured drug however used skillfully, will always prove to be a poison. Classics of Ayurveda do mention the hazards of drugs, which are not properly manufactured. Seers have prescribed specific processing techniques that remove harmful properties from these drugs. There is no sufficient evidence in the article to confirm the harmful nature of the formulation.

 Overall, it becomes clear from the manuscript that there is a serious need of looking into the drug regulating mechanisms in the country instead of blaming a system as such.

I confirm that I have read this submission and believe that I have an appropriate level of expertise to confirm that it is of an acceptable scientific standard, however I have significant reservations, as outlined above.

## References

[1] : Blood compatibility studies of Swarna bhasma (gold bhasma), an Ayurvedic drug. *Int J Ayurveda Res*.2011;2(1) : 10.4103/0974-7788.83183 14-22 10.4103/0974-7788.83183 21897638PMC3157103

[2] : Toxicity Study of Swarna Bhasma, an Ayurvedic Medicine Containing Gold, in Wistar Rats. *Toxicology International (Formerly Indian Journal of Toxicology)*.2015;22(3) : 10.22506/ti/2015/v22/i3/137618 10.22506/ti/2015/v22/i3/137618

[3] : Identification of Normal Blood Pressure in Different Age Group. *Medicine (Baltimore)*.2016;95(14) : 10.1097/MD.0000000000003188 e3188 10.1097/MD.0000000000003188 27057846PMC4998762

# Peer review: Reviewer response for version 1

**DOI:** 10.21956/wellcomeopenres.16470.r35563

**Published:** 2019-06-17

**Authors:** Kshirod Kumar Ratha

**Affiliations:** 1 National Research Institute of Ayurvedic Drug Development, Kolkata, West Bengal, India

The cases reported/discussed by the author are very serious. The authors tried to malign the Ayurveda system which is evident from the title of the manuscript it self,which need to be rectified. All natural drugs are not always safe. The medicines mentioned in the manuscript in puriya (packet form or unlabelled with out a valid prescription) are usually unethical and unlawful. Without a valid prescription and certificate of analysis of the drug it is very difficult to say they were Ayurvedic drugs.

 Also, without a valid prescription by a registered medical officer they can not be confirmed as Ayurvedic doctors rather the authors could labelled them as Quacks without any medical degree. In case 1, as the base line data of patient who had jaundice before the administration of drug was also not available, which makes it difficult to speculate liver injury caused by Ayurvedic drugs without any histopathology and drug analysis report. Hence, the link between the drug and liver failure can not be linked convincingly. Properly manufactured and prescribed in a therapeutic dose is always safe even in pediatric age group. Genuine Ayurvedic Metallic preparations also can be prescribed as they contains permissible metallic traces. Proper control on manufactuing and Quality control of any drug is the out look of the regulatory bodies of the state (1) Rauwolfia also in a small dose is very good for treating hypertension. The amount of Rauwolfia present and the reserpine amount required to cause the adverse effect needs to be highlighted. Because Rauwolfia contains so many alkaloids apart from Reserpine, which cause less possibility of adv. effect than Reserpine. (2) The combination of Rauwolfia with other drugs is usually mentioned in Ayurveda classics and only Rauwolfia is rarely prescribed in Ayurveda.

I confirm that I have read this submission and believe that I have an appropriate level of expertise to confirm that it is of an acceptable scientific standard, however I have significant reservations, as outlined above.

## References

[1] : Evidence-based safety and efficacy of Ayurvedic herbo-metallic preparations containing gold, iron, and mercury with special reference to pediatrics. *Medical Journal of Dr. D.Y. Patil University*.2017;10(3) : 10.4103/0975-2870.206579 10.4103/0975-2870.206579

[2] : Rauwolfia in the Treatment of Hypertension. *Integr Med (Encinitas)*.2015;14(3) :40-6 26770146PMC4566472

# Response

**Published:** 2019-07-29

**Authors:** Sudeep Adhikari

**Affiliations:** Patan Academy of Health Sciences, Nepal

**Thank you for your constructive comments.**

**The cases reported/discussed by the author are very serious. The authors tried to malign the Ayurveda system which is evident from the title of the manuscript itself which need to be rectified.**

**Answer**- The title of the manuscript has been revised as- ‘Adverse events with ayurvedic medicines- possible adulteration and some inherent toxicities’. The revised title does not intend to malign the Ayurveda system, but tries to raise the issues of adulteration and some inherent toxicities of medicines that have given bad name to the system of Ayurveda.

**All natural drugs are not always safe. The medicines mentioned in the manuscript in puriya (packet form or unlabelled without a valid prescription) are usually unethical and unlawful. Without a valid prescription and certificate of analysis of the drug it is very difficult to say they were Ayurvedic drugs.**

**Also, without a valid prescription by a registered medical officer they can not be confirmed as Ayurvedic doctors rather the authors could labelled them as Quacks without any medical degree.**

**Answer- **The traditional healers who claimed themselves to be ayurvedic practitioners prescribed the unlabeled puriyas and tablets to our patients. Analysis of the puriya drug taken by case 1 was not done. However the tablet taken by case 3 was analysed and steroid was found to be present. Hence we have raised the issue of adulteration in the name of ayurvedic medicines, giving bad name to the Ayurveda.

**In case 1, as the base line data of patient who had jaundice before the administration of drug was also not available, which makes it difficult to speculate liver injury caused by Ayurvedic drugs without any histopathology and drug analysis report. Hence, the link between the drug and liver failure can not be linked convincingly.**

**Answer- **The base line data of the first patient is not available. We have not performed histopathology and drug analysis report. So we are not sure if the drug contained harmful alkaloids, and if the worsening of the liver disease was due to disease process itself or the unlabeled drug that was given. We have mentioned this in our revised manuscript. Here we wanted to focus that at least the alternative drug our patient tried did not help in her liver problem as is generally believed by Nepalese population.

**Properly manufactured and prescribed in a therapeutic dose is always safe even in pediatric age group. Genuine Ayurvedic Metallic preparations also can be prescribed as they contains permissible metallic traces. Proper control on manufactuing and Quality control of any drug is the out look of the regulatory bodies of the state.**

**Answer- **The case 1 also received swarnabhasma (gold salt) for jaundice, and developed kidney injury. Heavy metals can be beneficial if used in permissible amount, but there are reports of presence of different heavy metals exceeding the maximum permissible amount in different ayurvedic preparations. This raises the issue of possible toxicities of these inherent constituents of ayurvedic medicines.

**Rauwolfia also in a small dose is very good for treating hypertension. The amount of Rauwolfia present and the reserpine amount required to cause the adverse effect needs to be highlighted. Because Rauwolfia contains so many alkaloids apart from Reserpine, which cause less possibility of adv. effect than Reserpine. The combination of Rauwolfia with other drugs is usually mentioned in Ayurveda classics and only Rauwolfia is rarely prescribed in Ayurveda.**

**Answer- **Although reserpine has safely been used in treating patients with hypertension in some studies in the form of Rauwolfia, the routine use of reserpine as antihypertensive agent has not been recommended by any of the current guidelines of allopathic medicine. Moreover, the ayurvedic preparation our patient was taking for hypertension did not have label regarding amount of reserpine in each tablet. So we have raised the issue about mentioning the exact amount of each chemical constituent in any ayurvedic product so that the patients have some idea about the substance and the quantity being taken.

# Peer review: Reviewer response for version 1

**DOI:** 10.21956/wellcomeopenres.16470.r35472

**Published:** 2019-06-05

**Authors:** Sanjib Kumar Sharma

**Affiliations:** 1 Department of Internal Medicine, B.P. Koirala Institute of Health Sciences, Dharan, Nepal

The Authors tried to explain a common scenario of the use of alternate medicine observed by the many clinicians (albeit not documented much) in Nepal. The case reported is of importance to clinical practice and written well.

 Ayurvedic medicines have been used in Nepal since the time immemorial. Ayurvedic and herbal medicines are known to cause varying degrees of drug-induced liver injury. Severe liver injury related to Ayurvedic and herbal medicines is studied to certain extent from the Indian subcontinent. Below are my comments for the note of the authors.  

 Case 1: The lady developed features of acute liver failure. Ingestion of gold formulation is known to cause hepatic injury by when the storage capacity of lysosomes exceeds ^1^. The parenteral preparation can, however, cause acute fulminating hepatic failure ^2^. Hepatotoxicity by pyrrolizidine alkaloids, often a composition in Ayurvedic medicine has been long recognized.

 Although the authors presume that the gold or other content of the consumed Ayurvedic medicine is likely to be the factor causing liver failure, the etiology of the liver injury is not clear in this case report. Histopathological study of the liver biopsy would have shed more light to understand the nature of the liver injury. Moreover, analysis of the available sample should have allowed knowing if proposed alkaloids were actually present in the Ayurvedic medicine consumed.  

 Case 2 – Reserpine is no more produced by pharmaceutical companies. It is implicated multiple side effects including postural hypotension, has not been tested as monotherapy in a clinical trial. However, reserpine had been used in large renal disease prevention program in India and was reported to have good blood pressure control without much adverse effect ^3^. A Chinese study of polypill contained low dose reserpine and had not shown more adverse effects ^4^. Similarly, reserpine was found to be safe in the treatment of cocaine dependence ^5^. In this clinical trial the safety results suggest that reserpine was safe and well tolerated by the participants.

 Case 3: The case exampled the common problem faced by clinical. Adulterated Ayurvedic medicine is a common issue in the Indian subcontinent. In 2011, Ayurvedic research center of KEM hospital Mumbai, India, reported out of the 244 Ayurvedic samples that were analyzed for steroid 96 samples were tested positive. About 18 samples had more than one steroid. This case report may help to create awareness among practicing clinician not aware of the problem or habit of looking into it.

I confirm that I have read this submission and believe that I have an appropriate level of expertise to confirm that it is of an acceptable scientific standard.

## References

[1] : Light- and electron-microscopical study of a case of gold salt-induced hepatotoxicity. *Hepatology*.1991;14(3) :422-5 1908434

[2] : Fatal hepatic necrosis associated with parenteral gold therapy. *Dig Dis Sci*.1988;33(8) :1025-9 313417610.1007/BF01536001

[3] : What we should do for chronic renal failure in India. *Journal of Mahatma Gandhi Institute of Medical Sciences*.2017;22(2) : 10.4103/jmgims.jmgims_34_17 10.4103/jmgims.jmgims_34_17

[4] : Long-term efficacy and tolerability of a fixed-dose combination of antihypertensive agents: an open-label surveillance study in China. *Clin Drug Investig*.2011;31(11) : 10.2165/11587390-000000000-00000 769-77 10.2165/11587390-000000000-00000 21671689

[5] : A double-blind, placebo-controlled trial of reserpine for the treatment of cocaine dependence. *Drug Alcohol Depend*.2007;91(2-3) : 10.1016/j.drugalcdep.2007.05.021 205-12 10.1016/j.drugalcdep.2007.05.021 17628352PMC10556850

# Response

**Published:** 2019-07-29

**Authors:** Sudeep Adhikari

**Affiliations:** Patan Academy of Health Sciences, Nepal

Thank you for your constructive comments.

 We have not performed histopathology and drug analysis report. So we are not sure if the drug contained harmful alkaloids, and if the worsening of the liver disease was due to disease process itself or the unlabeled drug that was given. We have mentioned this in our revised manuscript. Here we wanted to focus that at least the alternative drug our patient tried did not help in her liver problem as is generally believed by Nepalese population.

 Although reserpine has safely been used in treating patients with hypertension in some studies in the form of Rauwolfia, the routine use of reserpine as antihypertensive agent has not been recommended by any of the current guidelines of allopathic medicine. Moreover, the ayurvedic preparation our patient was taking for hypertension did not have label regarding amount of reserpine in each tablet. So we have raised the issue about mentioning the exact amount of each chemical constituent in any ayurvedic product so that the patients have some idea about the substance and the quantity being taken.
